# Acceptability and feasibility of using a blended quality improvement strategy among health workers to monitor women engagement in Option B+ program in Lilongwe Malawi

**DOI:** 10.1186/s12913-024-11342-z

**Published:** 2024-07-25

**Authors:** Wiza Kumwenda, Angela M. Bengtson, Shaphil Wallie, Tarsizious Chikaonda, Mitch Matoga, Agatha K. Bula, Jimmy Ba Villiera, Edith Kamanga, Mina C. Hosseinipour, Victor Mwapasa

**Affiliations:** 1 UNC Project-Malawi, Tidziwe Center, P/Bag A104, 100 Mzimba Road, Lilongwe, Malawi; 2 Department of Community and Environmental Health, Kamuzu University of Health Sciences, Blantyre, Malawi; 3 Department of Epidemiology, Emory University, Atlanta, GA USA; 4 Department of Medicine, University of North Carolina, Chapel Hill, NC USA

**Keywords:** Quality improvement, Acceptability, Feasibility, Engagement in Option B+

## Abstract

Option B + provides lifelong ART to pregnant and breastfeeding women with HIV to reduce mother-to-child transmission of HIV (eMTCT) and improve maternal health. The effectiveness of Option B + relies on continuous engagement, but suboptimal monitoring of HIV care hinders our measurements of engagement. Process mapping and quality improvement (PROMAQI) is a quality improvement strategy for healthcare workers (HCWs) to optimize complex processes such as monitoring HIV care. We assessed the acceptability and feasibility of the PROMAQI among HCWs and identified barriers and facilitators for PROMAQI implementation. A cross-sectional study using a mixed method approach was conducted from August 2021 to March 2022 across five urban health facilities participating in PROMAQI implementation n the Lilongwe district, Malawi. We assessed PROMAQI acceptability and feasibility at the end of the study. A 5-point Likert (1 = worst to 5 = best) scale tool was administered to 110 HCWs (*n* = 15–33 per facility) involved in PROMAQI implementationThese data were analysed using descriptive statistics Among the 110 HCWs, twenty-two (QI team (*n* = 11) and QI implementers (*n* = 11)) were purposively selected for in-depth interviews. Thematic analysis was conducted using deducted and inductive approaches. The theoretical framework for acceptability (TFA) was used to identify reasons for acceptability. The Consolidated Framework for Implementation Research (CFIR) was used to characterize the barriers and facilitators of PROMAQI implementation. HCWs recruited had a median age of 37 (32–43) years, 82.0% of whom were female. Most (42%) had completed secondary education, and 84% were nurses and community health workers. The median (IQR) acceptability and feasibility scores for the PROMAQI were 5 (IQR 4–5) and 4 (IQR 4–5), respectively. Reasons for high PROMAQI acceptability included addressing a relevant gap and improving performance. Perceived implementation barriers included poor work attitudes, time constraints, resource limitations, knowledge gaps, and workbook difficulties. The facilitators included communication, mentorship, training, and financial incentives. PROMAQI is a highly acceptable and feasible tool for monitoring engagement of women in Option B + . Addressing these barriers may optimize the implementation of PROMAQI. Scaling up PROMAQI may enhance retention in the Option B + program and facilitate eMTCT.

**Supplementary Information:**

The online version contains supplementary material available at 10.1186/s12913-024-11342-z.

## Contributions to the literature

- Process mapping and quality improvement (PROMAQI) is an acceptable and feasible strategy for monitoring women’s engagement.
- Mentorship, continuous communication between QI leads and QI implementers, and collaborative learning between QI teams and incentives are important for successful PROMAQI implementation.
- Successful implementation of the PROMAQI requires the protection of health workers who lead QI activities and the simplification of QI tools.

## Background

The World Health Organization (WHO) recommends universal lifelong antiretroviral therapy (ART) for pregnant and breastfeeding women living with HIV (PBW), known as Option B + , [1] as a strategy for eliminating HIV mother-to-child transmission (eMTCT) and improving maternal survival. The success of Option B + depends on the retention of PBWs in HIV care [2–4]. Retention is a challenge in most countries, including Malawi, where an estimated 20–25% of women disengage from Option B + at 12 months [5, 6]. Improving retention requires a multifaceted approach that first optimizes the process of tracking PBW engagement in HIV care [7, 8]

Optimal monitoring entails that frontline healthcare workers (HCWs) identify and characterize PBWs who inconsistently engage to HIV care in a timely manner to determine appropriate evidence-based interventions for retention. However, monitoring engagement is suboptimal because of misclassification of outcomes and missed opportunities [6, 9].

Quality improvement (QI) is a promising strategy for optimizing HIV care monitoring and improving HIV outcomes [10]. QI has been associated with improvements in ART uptake, adherence, and viral load suppression [11]. However, the application of QI to improve monitoring for PBW in Option B + is limited. Therefore, the authors implemented Process Mapping and Quality Improvement (PROMAQI), a blended QI approach that includes QI capacity building on a model for improvement (MFI) with a focus on process mapping, continuous QI mentorship, collaborative learning, structured feedback mechanisms, and QI resource support. Our study aimed to 1) evaluate the acceptability and feasibility of using PROMAQI as an implementation strategy for monitoring women’s engagement in the Option B + program among HCWs, including examining the association between HCW characteristics and implementation outcomes; 2) describe the reasons for its acceptability and feasibility after six months of implementation among HCWs; and 3) identify barriers and facilitators for PROMAQI implementation.

## Methods

### Study design

We conducted a cross-sectional study using mixed-method convergent approach in which the primary approach was quantitative, complemented by qualitative methods. A mixed-methods design was chosen to enhance the significance of the study by augmenting quantitative findings with the richness of qualitative data, thereby achieving a more in-depth understanding of the results [12, 13].

### Study setting and period

The study took place in five urban health facilities in Lilongwe, Malawi, with a combined catchment population of 700,000. These facilities offer antenatal care (ANC) and ART services together, catering to over 50,000 pregnant women annually, including approximately 2,000 women living with HIV [14]. High volume urban facilities are associated with increased LTFU and suboptimal monitoring [15, 16], PROMAQI was implemented from 01 August 2021 to 31 March 2022, in ANC or maternity clinics of these facilities.

### PROMAQI description

PROMAQI involved forming multidisciplinary QI teams within each healthcare facility, typically comprising 5–9 members selected from various HCW cadres responsible for Option B + monitoring. Team composition was determined by facility leadership or through consensus among HCWs. Each team included a chairperson providing leadership, a secretary managing communications, data collectors gathering change information, and monitors tracking implementation progress.

Support for QI teams came from facility leaders who served as QI team patrons (e.g., facility in charge, matrons), advocating for team needs. Researchers (study coordinator and principal investigator) served as QI mentors, offering training and guidance, while other HCWs (implementors) executed projects and provided feedback. QI teams underwent a 2-day initial training on MFI, emphasizing process mapping, followed by a refresher session two weeks into each project. Monthly mentoring sessions on QI methods were conducted, and QI teams and implementors met at least once a month for updates and feedback on the QI project. Peer-to-peer learning sessions on QI methods and monitoring engagement in Option B + were held at 3 and 6 months.

Researchers provided essential resources such as stationery, printing services, and meeting incentives for executing the QI method. They also supplied pre-designed QI journals to guide teams through each QI step, ensuring thorough documentation. Additionally, pre-designed QI meeting minute books supplemented this documentation effort. Patrons facilitated additional resources requested by QI teams for implementing change ideas in their projects.

### Study population and sampling

Our population included HCWs involved in Option B + service delivery and PROMAQI implementation. Each site provided a roster of HCWs involved in Option B + , totalling 401 across all sites. We randomly selected 110 HCWs at an estimated 95% confidence level and a precision level of ± 8%, assuming *p* = 0.05. The sample was stratified by cadre to allow adequate representation of all types of HCWs. We used Qualtrics sample size calculator for sample size calculation [17].

We purposively selected 22 HCWs from the 110 HCWs who were part of the QI leading team (11) or QI implementors (11) and were available throughout PROMAQI implementation. This was done to obtain comprehensive insights on the phenomenon of interest from both the deliverers (QI lead team member) and users (QI implementors). The sample size was determined to achieve data saturation because it aligns with a recognized benchmark for saturation of 9 to 17 participants [18].

### Data collection and management

Data collection was conducted for one month following the end of PROMAQI implementation. Before data collection, the study coordinator provided day-long protocol refresher training to research assistants (RAs) and generated a list of eligible HCW. RAs scheduled appointments at the convenience of the HCWs. The collection took place in a private facility location and was conducted in the preferred language (Chichewa or English) of the HCWs.

***For the quantitative aspect***, at enrolment, RAs collected participant characteristics: age, sex, education level, cadre, and years of experience. At 6 months, RAs administered a survey with adapted questions from the Acceptability of Intervention Measure (AIM) and Feasibility of Intervention Measure (FIM) [19]. Each had four Likert scale questions, with five-point responses ranging from 'completely disagree' (1) to 'completely agree’ (5). AIM assessed PROMAQI acceptability on the following aspects approve, appeal, liking, and welcome. Approval assessed agreement with implementation, appeal assessed perceived benefits or alignment with values, liking measured positive attitudes towards the strategy, and welcoming gauged openness or willingness to use the strategy. FIM assessed feasibility on the following aspects: implement, possible, doable, and ease of use [19]. Implement evaluates how easily the strategy could be integrated into workflow, possible assesses if it was realistic to implement, doable measured the practicality, and ease of use gauged user-friendliness and straightforwardness of the strategy. Data were entered into an electronic data capture system using Open Data Kit (ODK) and synchronized daily to a secure local server with encrypted tablets.

***For the qualitative aspect***, an experienced multilingual RA conducted semi-structured interviews using an in-depth interview guide (IDI) to explore PROMAQI acceptability and feasibility among HCWs. The IDI questions were guided by the Theoretical Framework for Acceptability (TFA), which encompasses seven constructs (affective attitude, perceived effectiveness, burden, ethicality, opportunity cost, intervention coherence, self-efficacy) [20]. The interviews were digitally recorded with participant consent and transcribed. Additionally, the RA directly translated Chichewa interviews into English during transcription. The PI ensured transcript quality by comparing content with audio recordings.

### Data analysis

#### Quantitative data

We used descriptive statistics (frequencies, proportions, means [standard deviations], and/or medians [interquartile ranges]) to describe participant characteristics and measure acceptability and feasibility at 6-month of implementing PROMAQI.

A chi-square test was conducted to examine the relationships between participant characteristics (including education, years of service, role, and site) and PROMAQI acceptability and feasibility. All analyses were carried out using Stata (version 14).

#### Qualitative data

We used NVivo and Microsoft Excel for qualitative data analysis. Thematic analysis was used [21]. An experienced qualitative researcher and the principal investigator coded a subset of the transcripts deductively and inductively. The codes were discussed and refined, a consensus was reached, a codebook was established, and the PI coded the remaining transcripts. The emerging reasons for PROMAQI acceptance and feasibility were mapped into TFA construct as main themes. Similarly, barriers and facilitators of PROMAQI were mapped into applicable consolidated framework for implementation research (CFIR) framework constructs. Independent qualitative expert reviewed the transcripts, codes, themes, and interpretations for validation.

#### Ethical considerations

The ethical approval to conduct the study was obtained from the ethics committee of the National Health Science Research Committee, Protocol #18/08/2137. All participants provided written informed consent before participating in the study.

## Results

### Characteristics of the study participants

Of the 110 participants enrolled (Table 1) who completed the PROMAQI assessment at 6 months, most (78%) were nurses and health surveillance assistants/counsellors (HSAs), predominantly (82%) were female, and the median age was 37 years. Over two-thirds had worked at the facility for more than 5 years.

**Table 1:** Characteristics of study participants

Variable	Total
	***N*** ** = 110**
Age, median (IQR)	
	37(32–43)
Gender (n, %)	
Male	20 (18.0)
Female	90 (82.0)
Education Level (n, %)	
Secondary certificate	46 (41.8)
Professional certificate	12 (10.9)
Diploma	38 (34.6)
Degree	14(12.7)
Cadres (n, %)	
Data	18 (16.4)
Nurse	41 (37.2)
Clinician	6 (5.4)
Health Surveillance Assistants/Counsellor	45 (41.0)
Duration at current role (years) (n, %)	
0–4	36 (32.7)
5–9	25 (22.7)
10–15	29 (26.4)
> 15	20 (18.2)

### Quantitative findings

#### HCWs’ perceptions of the acceptability and feasibility of the PROMAQI

**Fig. 1:**
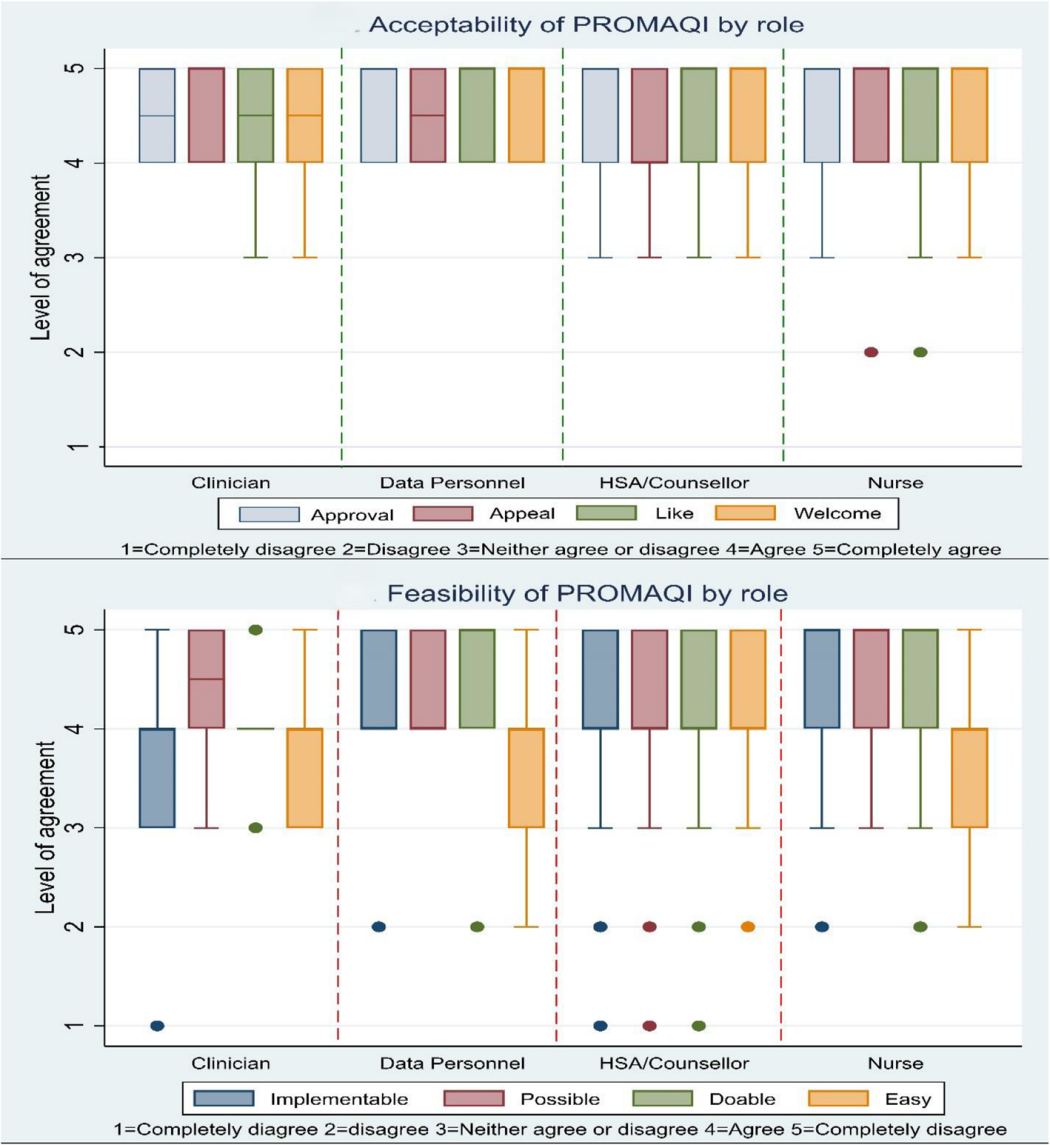
PROMAQI acceptance and feasibility by role at 6 months of implementation

PROMAQI was rated as highly acceptable (median score 5, IQR 4–5) and feasible (median score 4 (IQR 4–5)) among different cadres (Fig. 1). While both acceptability and feasibility were scored highly, the feasibility scores were lower compared to acceptability scores (Table 2).

Table 2 shows that roughly one-fifth of HCWs were neutral or disagreed about PROMAQI feasibility, with the majority of HCWs (30%) being concerned with ease of use, followed by doability, implementability, and possibility. Among the subset of HCWs that were concerned with ease of use (Table 3), our results showed significant differences were detected across sites (χ^2^(12) = 21.20, *p* = 0.05). However, there was no significant variation in opinions based on education, years of service, or role.

### Qualitative findings

#### Reasons for PROMAQI acceptance

PROMAQI was acceptable among participants and the reasons for the acceptance among QI lead team members and QI implementers were similar. Using TFA constructs, the reasons are outlined below.

#### Affective attitude and ethicality

All participants exhibited a positive attitude towards PROMAQI, believing it would enhance care quality, align with their values, provide educational value, improve work performance, and enhance teamwork.

*“I feel good because it was a learning process that helped myself to improve on the delivery of services especially to women who are HIV positive when they come to our facility... am a midwife, … it was an obvious thing that I need to participate”. Nurse, site 2, QI implementer*

#### Burden and opportunity cost

The implementation of the PROMAQI was not viewed as burdensome and was considered to be part of HCW tasks and in line with standard practice. However, some participants noted that undertaking these tasks demands sacrificing personal time, especially for QI leaders.

*"It (QI Project tasks) did not disturb any activity, or any interest because it was part of the job what I was doing each and every day. Therefore, it did not disturb but it helped to improve especially on the challenge that I was saying about the punctuality, … it helped to improve in reporting at good time at the facility" Nurse, 40 years old, QI implementer, Site 2*

*“Sometimes, I would have to come for a meeting when I could be off duty … or sometimes we had to have meetings even after knocking off time … in a way it interfered with my priorities because maybe I would want to be home” Nurse, 27 years, QI lead member, Site 3*

### Intervention coherence and self-efficacy

Participants understood PROMAQI well, highlighting the purpose of the QI projects involved, PROMAQI components used and challenges, its integration into their work, and differentiated the PROMAQI approach to other QI projects.

*"I have participated (QI) but it was not about PMTCT, it was about the whole facility whereby we were supposed to improve things like infection prevention, nursing standards, things like those... we didn’t have process mapping to identify the root causes of the problems... With this QI because of the process of identifying the problem, we were able to address the problem." Nurse, 55 years, QI lead member, site 5*

Participants' grasp of PROMAQI and its activities was linked to their task performance. Understanding the tasks served as both an enabler and motivator for participants, who expressed confidence in their QI skills and outlined plans to expand implementation or continue the project post-study. However, concerns were raised about QI journals and the limited number of HCWs proficient in developing QI projects independently, posing challenges for scalability and sustainability.

*“We need more people to be oriented in this method, this QI thing, because I feel that many people they truly don’t understand. They can help you in implementation (QI project), but they don’t truly understand how to come up with the project for QI.” Nurse, 32 years, QI lead member, site 1*

### Perceived effectiveness

Participants viewed PROMAQI effective based on its success, citing improved teamwork, enhanced service quality, changes in work practices, achievement of QI project goals, and PROMAQI task completion.

*"…. It was a success because … we managed to carry out all the PDSAs that were planned. Although looking at the goal of the project, we are below the target" Nurse, 33 years old, QI team member, Site 4*

### Barriers and facilitators of PROMAQI implementation

Below are key barriers and facilitators mapped into applicable CFIR domains and constructs:

1. Inner setting
  - Networks and communication: Participants emphasized the importance of continuous communication to help HCWs understand and engage in the implementation process, fostering a positive attitude towards the strategy. They recommended orientation, presentations, and regular meetings for evaluation and experience sharing.
    “What should happen is that there should be regular meetings so that we can be evaluated on where we are doing right or wrong so that everyone should be able to share the experiences...” Data personnel, 39 years, QI implementer, Site 3
  - Implementation climate: Participants noted challenges due to HCWs' poor attitudes and work practices, including uncooperativeness like late attendance or no-shows. Additionally, some participants were concerned that some HCWs inherently lacked the initiative.
  - Availability of resources: Participants identified the time needed to lead QI activities during PROMAQI implementation as a challenge. Additionally, they anticipated difficulties post-study related to the availability of HCWs with QI skills, access to mentors as per the PROMAQI design, and basic resources required for QI activities, including stationery and printing services.
    “It would need some resources because, at times, as a facility, we tend to lack some resources. For example, in our QI project, when we were capturing data, we had to use hardcovers. Therefore, such materials, sometimes, … we do not have the capacity to have those.” Nurse, 27 years old, QI team member, Site 3
  - Organizational incentives and rewards: Participants stressed the need for incentives or reimbursements in PROMAQI implementation as they prevent HCWs from using personal resources for QI activities. Additionally, they motivate participation, as seen with lunch allowances during meetings.
    “What you need to consider first is the issue of allowances because for someone to leave his/her duty station, what comes to mind first is the allowance that he/she is going to receive. This is a motivation.” Data personnel, 41 years old, QI implementer, Site 2
    One participant emphasized the need to clarify the purpose of incentives, as some prioritize them over the actual work. Additionally, there was concern that incentives could cause discontent among healthcare workers, as some believed QI leads received money while others did the work.
2. Intervention characteristics
  - Relative advantage and design and quality: Some participants found that the role of QI mentors in PROMAQI was distinct from that in other QI initiatives; it enhanced their competence and confidence in the application of QI. Some expressed leadership training and development of transition plans for QI leaders.
    “there are some NGOs that are also doing the same thing [QI] but what they do is they come teach you...after that they will come after six months, only to ask, have you done your project? Mmmh you see, it’s hard for people to understand but I can say the way you were doing it, having a facilitator [QI mentor] always there to review maybe after three or four weeks, how we did it [QI procedures] and provided guidance. This way, … people learn, and they can stand on their own. As for me now, and with my friends, I can say, we can stand and even teach other people” Nurse, 32 years, QI lead member, site 1
  - Complexity: The components of the PROMAQI that were perceived to be difficult to implement were man-management and the use of QI workbooks or journals. Participants largely described the process of completing a QI workbook or journal as difficult and tedious. Additionally, the journal was not fully understood and recommended for inclusion in the training plan.
    “First, we should think about the journal…first of all, people should be trained to know how to fill the journal. Most of us were not aware of how to fill out the journal. Therefore, if you are scaling to other facilities, consider training them on how to fill the journal.” Data personnel, 27 years, QI lead member, site 2

## Discussion

Our study revealed that for frontline HCWs, PROMAQI is a highly acceptable and feasible tool for monitoring Option B + engagement among women in large urban health facilities. Although the overall feasibility was high, one-fifth of HCWs expressed reservations about the strategy, varying by site. The key reasons for its acceptability included the belief that it could improve the quality of care, enhance skills, involvement in decision making, and seamlessly integrate with one’s roles. The facilitators of implementation were mainly continuous engagement between QI leaders and QI implementers, QI mentorship, collaborative learning, and incentives. Barriers/demotivating factors included time constraints, limited resources, difficulties in completing QI journals, and knowledge gaps for QI project development.

The high acceptability of PROMAQI found in this study was not surprising due to its characteristics, which centered on QI capacity building, QI mentorship, embracing collaborative learning, and having QI resource support. Previous studies have highlighted that these elements in each right or combined were embraced positively by participants in various QI initiatives [22–24]. Acceptance could be attributed to the 2016 Ministry of Health Malawi MoH effort to revamp QI structures and systems by establishing a QI directorate. The directorate provides strategic leadership and coordination of quality management initiatives across the health sector. Facilities are required to have QI teams that promote QI [25, 26]. PROMAQI acceptance aligned with the notion highlighted in Eswatini and Swedish studies that improvement projects do well where HCWs feel a sense of belonging, autonomy, professional competence, and a clear social goal [27, 28]. Acceptance is high if the project is easy and clearly integrates QI project tasks into daily routine tasks; otherwise, it hinders program implementation [27]. This positive position of HCWs suggests that PROMAQI can be easily used over time, as acceptance is a crucial precursor for successful implementation and sustainability [29].

The feasibility of the PROMAQI was generally positive, similar to other QI approaches [30]. However, some reservations were made about the process of journal documentation. HCWs often view information management tasks as a burden [31, 32]. Poor tool design or the complexity of QI measurements can adversely affect documentation [32, 33]. Exploring options such as electronic or standardized paper QI journals could simplify the process and boost usage [34]. Furthermore, the integration of continuous training for the tool is needed in the sustainability plan to enhance the understanding and utilization of the tools, which is critical to data use.

Similar to our study, previous research on QI has highlighted challenges such as limited time, expertise, and operational resources [27, 35, 36]. Addressing these issues is important, and one possible solution is to allocate protected time for QI initiatives to prevent fatigue and maintain motivation. This allocation may demonstrate the organization's recognition and support for the team, contributing to the initiative's success [37]. Additional strategies include on-going competency training on QI methodology, incorporation of QI in HCW training curricula, streamlining the tools and making use of technology-supported job aids, and performance support tools [33, 38, 39]. Furthermore, the government may provide targeted funding to facilities for the implementation of structured QI and continue collaborating with partner organizations for support [40] as long as the improvement goals are aligned. However, it is critical to assess the cost implications of implementing these strategies.

Ongoing QI mentorship, support from partner organizations, incentives, and regular communication were found to be facilitators of PROMAQI implementation, and these factors were not peculiar to our QI approach [34, 41, 42]. Strengthening partnerships and fostering collaboration are crucial for a supportive QI implementation environment, allowing diverse skills and resources to be leveraged for innovative solutions and improved outcomes. The structured communication process for obtaining feedback in PROMAQI enabled understanding of the project and its progress. Incentives promoted participation and dedication to PROMAQI activities; incentives increased job satisfaction, which motivated workers to perform well. However, defining the purpose of incentives is needed to prevent misunderstandings that may negatively affect morale, thereby affecting implementation [37, 43]. Overall, the barriers and facilitators suggest that institutional organizational factors are more salient than individual or external factors for QI implementation. The attributes of each organization’s context inherently differ, thus explaining the QI implementation variations [44, 45].

### Limitations

The study provides a general representation of urban health facilities in Malawi. However, self-reported data may be susceptible to bias or social desirability effects. To mitigate this bias, we employed a trained independent RA, used validated tools with good psychometric properties and good usability, and checked Cronbach's alpha to ensure internal consistency.

## Conclusion

PROMAQI was highly acceptable and feasible among HCWs for monitoring engagement and retention outcomes in the Option B + program in high-volume facilities in Malawi. However, addressing feasibility concerns requires strategic modifications. Key factors that could influence PROMAQI adoption and long-term success include resources, time, and expertise, emphasizing the need for adequate funding, human resource planning, and continued support for the successful implementation of QI projects. This study provides crucial insights into QI project implementation in resource-limited settings, emphasizing organizational factors, resource adequacy, and simplified QI tools for effective documentation. Further research is needed to assess the cost-effectiveness of the scalability and sustainability of the PROMAQI and its impact on patient outcomes.

PROMAQI's innovation lies in its holistic approach, integrating multidisciplinary teams, clear leadership, dual support systems, comprehensive training with process mapping, robust documentation tools, and structured feedback mechanisms. This approach could enhance sustainable QI practices by fostering a culture of continuous improvement and collaboration, thereby enhancing healthcare quality and patient outcomes. Notably, PROMAQI's emphasis on process mapping facilitates systematic analysis and optimization of healthcare processes, distinguishing it as a valuable model for enhancing operational efficiency and quality in healthcare settings.

### Supplementary Information

Supplementary Material 1: Table 2 Distribution of PROMAQI acceptability and feasibility.

Supplementary Material 2: Table 3 Distribution of PROMAQI perceived ease of use.

Supplementary Material 3.

## Data Availability

The dataset (which includes individual transcripts) is not publicly available due to confidentiality policies but are available from the corresponding author on reasonable request.

## Abbreviations

ANC: Antenatal Care Clinic
ART: Antiretroviral therapy
CFIR: Consolidated Framework for Implementation Research
eMTCT: Elimination of mother-to-child transmission of HIV
HCW: Healthcare worker
HSA: Health Surveillance Assistance/Counsellor
MFI: Model for Improvement
PBW: Pregnant and Breastfeeding Women
PDSA: Plan Do Study Act
PROMAQI: Process Mapping and Quality Improvement
QI: Quality improvement
RA: Research Assistant
TFA: Theoretical Framework for Acceptability
WHO: World Health Organization
