# Supplementary material for: Acceptability and feasibility of using a blended quality improvement strategy among health workers to monitor women engagement in Option B+ program in Lilongwe Malawi
Source: BMC Health Serv Res. 2024 Jul 25;24:842. doi: 10.1186/s12913-024-11342-z (PMC11282652; doi:10.1186/s12913-024-11342-z)
Supplement: Supplementary file 1 — Supplementary Material 1: Table 2 Distribution of PROMAQI acceptability and feasibility. [file 12913_2024_11342_MOESM1_ESM.docx]

**Table 2 Distribution of PROMAQI acceptability and feasibility**

| **Distribution of PROMAQI acceptability and feasibility (*n*=110)** | | | | | | | |
| --- | --- | --- | --- | --- | --- | --- | --- |
|  | **Frequency (%)** | | | | |  |  |
|  | Completely Disagree | Disagree | Neutral | Agree | Completely agree | Dichotomous rating | Cronbach's alpha |
| Acceptability |  |  |  |  |  | 96% | 0.79 |
| approval | 0 | 0 | 4 (3.64) | 42 (38.18) | 64 (58.18) |  |  |
| appeal | 0 | 1(0.91) | 3 (2.73) | 48 (43.63) | 58 (52.73) |  |  |
| Like | 0 | 1 (0.91) | 5 (4.55) | 35 (31.82) | 69 (62.73) |  |  |
| Welcome | 0 | 0 | 4 (3.64) | 30 (27.27) | 76 (69.09) |  |  |
| Feasibility |  |  |  |  |  | 84% | 0.76 |
| implementable | 2 (1.82) | 4 (3.64) | 9 (8.18) | 44 (40.00) | 51 (46.36) |  |  |
| possible | 1 (0.91) | 1 (0.91) | 6 (5.45) | 42 (38.18) | 60 (54.55) |  |  |
| doable | 1 (0.91) | 4 (3.64) | 11 (10.00) | 37 (33.64) | 57 (51.82) |  |  |
| easy | 0 | 8 (7.25) | 23 (20.91) | 43 (39.09) | 36 (32.73) |  |  |
