# Supplementary material for: Acceptability and feasibility of using a blended quality improvement strategy among health workers to monitor women engagement in Option B+ program in Lilongwe Malawi
Source: BMC Health Serv Res. 2024 Jul 25;24:842. doi: 10.1186/s12913-024-11342-z (PMC11282652; doi:10.1186/s12913-024-11342-z)
Supplement: Supplementary file 2 — Supplementary Material 2: Table 3 Distribution of PROMAQI perceived ease of use. [file 12913_2024_11342_MOESM2_ESM.docx]

**Table 3 Distribution of PROMAQI perceived ease of use**

| **Distribution of PROMAQI perceived ease of use (*n*=110)** | | | | | | | |
| --- | --- | --- | --- | --- | --- | --- | --- |
|  | | **Frequency (%)** | | | |  |  |
|  | | Disagree | Neutral | Agree | Strongly | **χ2** | **P** |
| Site | |  |  |  |  | 21.1978 | 0.048 |
| Site 1 | 3 (9.09) | | 3 (9.09) | 15 (45.45) | 12 (36.36) |  |  |
| Site 2 | 0 | | 3 (18.75) | 6 (37.50) | 7 (43.75) |  |  |
| Site 3 | 2 (11.11) | | 10 (55.56) | 5 (27.78) | 1 (27.78) |  |  |
| Site 4 | 2 (7.14) | | 5 (17.86) | 10 (35.71) | 11 (39.29) |  |  |
| Site 5 | 1 (6.67) | | 2 (13.33) | 7 (46.67) | 5 (33.33) |  |  |
| Role |  | |  |  |  | 8.2004 | 0.514 |
| Clinician | 0 | | 2 (33.33) | 3 (50) | 1 (16.67) |  |  |
| Data Personnel | 2 (11.11) | | 4 (22.22) | 8 (44.44) | 4 (22.22) |  |  |
| HSA/Counsellor | 3 (6.67) | | 8 (17.78) | 13 (28.89) | 21 (46.67) |  |  |
| Nurse | 3 (7.32) | | 9 (21.95) | 19 (46.34) | 10 (24.39) |  |  |
| Education |  | |  |  |  | 7.4801 | 0.058 |
| Certificate or below | 4 (8.33) | | 8 (16.67) | 14 (29.17) | 22 (45.83) |  |  |
| Diploma or higher | 4 (6.45) | | 15 (24.19) | 29 (46.77) | 14 (22.58) |  |  |
| Years of service |  | |  |  |  | 11.2726 | 0.257 |
| 0-5 years | 4 (11.11) | | 9 (25.00) | 14 (38.89) | 9 (25.00) |  |  |
| 5-9 years | 4 (16.00) | | 4 (16.00) | 11 (44.00) | 6 (24.00) |  |  |
| 10-15 years | 0 | | 5 (17.24) | 12 (41.38) | 12 (41.38) |  |  |
| >15 years | 0 | | 5 (25.00) | 6 (30.00) | 9 (45.00) |  |  |
