# Supplementary material for: Acceptability and feasibility of using a blended quality improvement strategy among health workers to monitor women engagement in Option B+ program in Lilongwe Malawi
Source: BMC Health Serv Res. 2024 Jul 25;24:842. doi: 10.1186/s12913-024-11342-z (PMC11282652; doi:10.1186/s12913-024-11342-z)
Supplement: Supplementary file 3 — Supplementary Material 3. [file 12913_2024_11342_MOESM3_ESM.docx]

**Acceptability and Feasibility Survey**

**Demographics**

**Site:** ________________

**Participant ID**: ___________________ **Date of interview** ___________

**QI role:**  part of QI team _(0)_  participated in QI activities _(1)_

**Vignette**

The purpose of this survey is to assess acceptability and feasibility of the strategy called PROMAQI which stands for Process Mapping and Quality Improvement. The strategy was designed to help health workers to improve the process of monitoring of women engagement to PMTCT care. The QI project has been running for 6 months at this facility. In this survey, you will be asked whether or not you agree or disagree based on a scale of 1 to 5. A grade of 1 means that you completely disagree while a grade of 5 means that you completely agree. Please remember that these questions are not a test but are only meant to gauge how you felt about the intervention. There are no right or wrong answers. Please answer the question with honesty.

**Acceptability**

|  | Completely disagree | Disagree | Neither agree nor disagree | Agree | Completely agree |
| --- | --- | --- | --- | --- | --- |
| 1. Using QI as a way to improve the process of monitoring women engagement meets my approval | ➀ | ➁ | ➂ | ➃ | ➄ |
| 1. Using QI as a way to improve the process of monitoring women’s engagement was appealing to me | ➀ | ➁ | ➂ | ➃ | ➄ |
| 1. I liked how QI was used in the process of monitoring women’s engagement | ➀ | ➁ | ➂ | ➃ | ➄ |
| 1. I welcome the idea of continuously using QI in the process of monitoring women’s engagement | ➀ | ➁ | ➂ | ➃ | ➄ |

**Feasibility**

|  | Completely disagree | Disagree | Neither agree nor disagree | Agree | Completely agree |
| --- | --- | --- | --- | --- | --- |
| 1. Using QI in the process of monitoring women’s engagement seems implementable | ➀ | ➁ | ➂ | ➃ | ➄ |
| 1. Using QI in the process of monitoring women’s engagement seems possible | ➀ | ➁ | ➂ | ➃ | ➄ |
| 1. Using QI in the process of monitoring women’s engagement seems doable | ➀ | ➁ | ➂ | ➃ | ➄ |
| 1. Using QI in the process of monitoring women’s engagement seems easy | ➀ | ➁ | ➂ | ➃ | ➄ |

**ACCEPTABILITY AND FEASIBILITY IN-DEPTH INTERVIEWS GUIDE SECTION**

**Instructions for Interviewer**

Your job as an interviewer is to facilitate honest and detailed responses about what the interviewee actually believes about any particular response to the questions below. This is not an exam for participants agreeing to be interviewed. There are no “right” or “wrong” answers. It is permissible to ask a participant to clarify a response if you do not understand. However, do not seek unnecessary clarification, causing the nature of the original response to change substantively. While the discussion should feel natural, avoid providing too much of your own personal insight, which may lead or sway a participant to reach your own pre-determined conclusion. Your job is to motivate the participant to expand on their own ideas, and allow them to reach conclusions on their own.

The next set of questions below have been designed as to not solicit simple “yes” and “no” answers, but are open-ended in such a way that participants can answer them as they see fit, given their own experience and knowledge of the question. Your role as a guide is to keep participants from straying off topic. To accomplish this, you may ask for details, stories, anecdotes, descriptions of setting, opinions, attitudes, and perceptions about responses to answers that are already on topic. Avoid repeating a question, which, you feel, has already been adequately addressed. Thus, it is not necessary to ask each and every question in the IDI guide in the sequence that has been provided.

**Vignette**

We would like to understand your point of view regarding the acceptability and feasibility of using quality improvement as a method for improving the process of monitoring women engagement to Option B+ services.

**Acceptability and Feasibility Guide:**

|  | QI Lead team member: These are member of the QI team selected to lead QI activities: | QI activities participant: The are members that were implementing QI activities: |
| --- | --- | --- |
| **Affective Attitude** | 1. What do you know about quality improvement? 2. What QI project were you working on? 3. What made you decide to participate in this QI project? 4. What was your experience in participating this QI project? 5. How did you feel about the implementation of this QI project?    - How do you feel about the method used to come up with this QI Project?    - Was there anything in particular that you liked or not liked about QI project      - What about the method used to come up with this project, was there anything particular that you liked or disliked?    - What expectations did you have with this QI project? Which of the expectations were met? 6. What made this QI Project acceptable or not acceptable to you?    - What about your colleagues, what areas do you feel were acceptable or not acceptable to them?    - If not acceptable, what should happen to make it more acceptable? | 1. What do you know about quality improvement? 2. In general, how do you feel about participating in any QI project activities? 3. What do you know about the QI project that was being conducted? 4. What made you participate or not to participate to the efforts presented by QI team? |
| **Burden** | 1. What areas did you find easy or difficult to implement in this QI project?    - Was the method for coming up with the QI project easy or not easy? If yes, which areas of the method did you find it easy? If not what areas did you not understand and how can we improve? 2. In what way did the QI project interfere with your work priorities and interest? | 1. How easy or difficult was it to participate in QI activities presented by the QI team? 2. In what way did the QI project activities disturb your work priorities and interest |
| **Ethicality** | 1. How did the QI project fit in with the way you do your job? 2. What changes did you make to ensure that this QI project fits with how things should be done or how you work? | 1. How does the QI project align with the way you do your job? 2. What changes did you make in order to align QI activities with the way you do your job? |
| **Intervention coherence** | 1. To what extent do you understand the process of coming up with a QI project? 2. How was the process of coming up with the QI program explained to you? 3. How were your specific role and responsibilities explained to you? 4. What level of input did you have in defining your responsibilities? 5. What should be done to improve your understanding of coming up with the QI Project | 1. To what extent did you understand what the QI team lead wanted 2. How were the activities explained to you? 3. What should be done in order to improve the understanding of the QI project activities? |
| **Opportunity cost** | 1. In your opinion, what did this QI project contribute to your day to day work? 2. What benefits did the QI project bring at this facility? 3. What were you able to forego in order to use the QI method to monitor engagement of women to PMTCT care? 4. What values did you use or were needed for you to conduct the QI project appropriately? | 1. What benefits does the QI project bring to your day to day work? 2. What tasks did you let go in order to include QI activities in your work? |
| **Perceived Effectiveness** | 1. Was the QI project a success? Please explain why    1. Will QI be continued? Please explain why    2. Will QI be changed? Please explain why 2. What difference did the QI project bring compared to how things were done previously at this facility? 3. What do you think would be helpful for us to consider if it is to be scaled up across other facilities? | 1. What difference did the QI project bring compared to how things were done previously at this facility? 2. What do you think would be helpful for us to consider if it is to be scaled up across other facilities? |
| **Self-Efficacy** | 1. What have you learnt from this QI project? 2. In a scenario that you have all the required resources, to what extent do you feel confident that the facility can conduct a QI project like this on its own? 3. What should be done to improve your capability to run a QI project? 4. In your opinion, what made your colleagues to support or not support the QI project? | 1. In your opinion, do you foresee the QI team continuing coming up with QI projects? Why do you think so? 2. Do you think the QI activities will continue to be supported you and your colleagues? Why do you think so? 3. What made you and your colleagues to support or not support the QI team? |
